# Cultural transmission of civic attitudes

**DOI:** 10.1186/s40064-016-2616-4

**Published:** 2016-06-30

**Authors:** Daniel Miles-Touya, Máximo Rossi

**Affiliations:** RGEA, Facultad de Ciencias Económicas y Empresariales/Universidade de Vigo, As Lagoas-Marcosende s/n, 36310 Vigo, Spain; Departamento de Economía, Facultad de Ciencias Sociales/Universidad de la Republica, Constituyente 1502, 11200 Montevideo, Uruguay; Center for Inter-American Policy and Research (CIPR), Tulane University, New Orleans, USA

**Keywords:** Returns to education, Cultural transmission, I20, H80

## Abstract

In this empirical paper we attempt to measure the separate influence on civic engagement of educational attainment and cultural transmission of civic attitudes. Unlike most of the previous empirical works on this issue, we are able to approximate the cultural transmission of civic attitudes. We observe that civic returns to education are overstated when the transmission of civic attitudes is ignored. Moreover, the transmission of civic attitudes significantly enhances civic involvement and reinforces civic returns to education. Our findings are in line with the proposals of civic virtue theorists or grass movements who suggest that citizenship education should be included in the compulsory school curricula since, if not, families or local communities will only transmit their particular view of the world.

## Background

The social benefits of education constitute a well-known empirical regularity: countries or regions with higher educational attainment enjoy lower crime rates, better health outcomes or improved performance across different socioeconomic measures. As Putman (2001) argues, education is one of the most important predictors of many forms of social participation, from voting and associational membership, to chairing a local committee, hosting a dinner party, or giving blood. This strong and positive correlation between educational attainment and civic outcomes has supported the view that education is effective at promoting civic behavior.

However, several authors have argued that these conventional correlations may overestimate social benefits of education because schooling and civic outcomes are simultaneously influenced by a variety of unobservable traits specific to the environments within which individuals are reared. There is evidence that the intergenerational or cultural transmission of civic attitudes during adolescence is relevant in explaining both educational attainment and adult civic behavior. On the one hand, children of civically engaged parents are expected to do better in school and to achieve higher levels of educational certification than children reared in other environments. On the other hand, parents' attitudes and political engagement shape their children’s worldview and also their civic behavior later in life. In other words, the positive correlation between educational attainment and civic behavior could be the result of the transmission of civic attitudes during adolescence (Beck and Jennings 1982; Rosenstone and Hansen 1993; Picketty 1995; Gutmann 1999; Flanagan 2003; Dee 2004; Kam and Palmer 2008; Sondheimer and Green 2010).

In this empirical paper we seek to shed some light on the separate influence on civic engagement of the transmission of civic attitudes and of educational attainment using Spanish data. We benefit from the fact that the survey we use reports information about the civic engagement environment surrounding the respondent’s adolescence.

In previous studies, the absence of data to approximate the transmission of civic attitudes have made it particularly difficult to separate the additional schooling effect from the transmission of civic attitudes effect in order to assess their relative importance in enhancing civic engagement. Hence, most of the papers which analyze this issue have been concerned with identifying only the educational attainment effect on adult civic engagement (Campbell 2006). Basically, two empirical strategies have been followed to circumvent the problem of not observing the transmission of civic attitudes. Some papers have assumed that the individual unobservable differences in cultural transmission of civic attitudes could be accounted for by including a comprehensive set of explanatory variables (similar to selection on observables or unconfoundedness matching approach). For example, under this approach Nie and Hollygus (2001) confirm that civic returns to education are both positive and statistically significant, suggesting that education is effective at promoting civic behavior. Nonetheless, these additional variables might not truly capture the unobserved transmission of civic attitudes, leaving the problem of confoundedness unresolved.

Other recent studies have relied on a standard instrumental variables approach, which requires an exogenous change in educational attainment and linearly separable unobservable. Using the proximity of colleges or differences in state child labor laws (Dee 2004) or the change in number of compulsory years of schooling (Milligan et al. 2004) as instrumental variables, these authors find evidence that additional schooling for the would-be dropouts significantly increases the probability of voting and enhances measures of civic-mindedness such as newspaper readership and free speech in the United States and United Kingdom. Nevertheless, using a similar instrumental variable, Siedler (2010) finds no evidence that additional schooling enhances civic outcomes in Germany, and neither do Kam and Palmer (2008), who do find clear evidence that voting behavior differ by college attendance. Likewise, Berinsky and Lenz (2011) find little evidence of a causal relationship between educational attainment and political participation for those individuals induced to increase their educational levels by the Vietnam draft. In other words, these papers based on instrumental variables report mixed evidence on whether additional schooling results in higher levels of civic involvement during adulthood.

From the political science point of view, disentangling the influence of the transmission of civic attitudes and the additional schooling effect on civic involvement has grown more important in recent years. While all sides agree that the stability of democratic institutions and the effectiveness of public policies depends to a great extent on the quality and attitudes of citizens, there is an important disagreement on who is mostly responsible for teaching civic values, schools or families (Kimlycka and Norman 1994; Gutmann 1999; Sandel 2010). The dominant trend since the mid of the last century has embraced the view that governments intervention in education does not extend to the teaching of citizenship or moral education, that being the role of families. Following this trend, education systems in most developed countries evolved from a vision of education for civic virtue to a vision of education as a response to market needs, leaving the education of civic values to the family (Labaree 2010).^1^ Under this view, most European countries’ post-war educational policies were not designed to stimulate an active involvement of citizens in civil society or political decision making (Roche 1992; Giddens 1999).

Civic virtue theorists have argued that relying solely on families to teach civic values could lead to the exclusive promotion of the predominant values or worldview among better-educated individuals. Parents who stress civic responsibilities also encourage more schooling, and hence better-educated individuals are a particular selection of individuals in the population. Moreover, parents and communities tend to transmit only their worldview and not alternative viewpoints (religion, race dominance, winners-take-all, etc.). Therefore, when the school system does not expose students to alternative points of views, the better-educated individuals will tend to reproduce or promote their particular viewpoint, e.g. those stressed by their parents or communities. In other terms, civic virtue theorists advocate that the educational system should expose students to different civic and moral values than that of their families. For example, Gutmann (1999) remarks that “*education for democratic citizenship… 'equip children with the intellectual skills necessary to evaluate ways of life different from that of their parents*'” (Kimlycka and Norman 1994, pp. 366–367). Therefore, understanding the different contributions of transmission of civic attitudes and educational attainment on civic engagement could shed some light on this discussion.

Notice that, despite a significant increase in educational attainment in developed countries, recent evidence suggest that an increasing proportion of the population of these countries show apathy toward political affairs, observed in the diminishing voter turnout, in membership in political parties, civic associations or in the involvement in social affairs. In part, these evidence has triggered, since the early 2000, the interest of policy-makers and democracy advocates in most developed countries in order to search ways to enhance democratic attitudes and civic habits. One of the main channels chosen to achieve these targets is through formal schooling, by introducing citizenship education in the compulsory school curricula, e.g., Portugal or UK curricula reorganization in 2001 or 2002, respectively, with the introduction of citizenship education issues (Ribeiro et al. 2012). Among others, the European Commission has launched the program “Citizens for Europe” to promote civic participation and a stronger sense of citizenship by motivating changes in the high school curricula of its country members (Eurodyce 2005), e.g., Kerr et al. (2010) presents evidence of the priority assigned to these issues by EU country. In the United States, there is a debate at state levels of how to adequately implement citizenship educational policies in schools given the downward trend in voter turnout or membership in political or civic associations (Educational Commission USA 2004). In Canada the discussion is on the type of education that is necessary to uphold a diverse and democratic country (Hebert and Sears 2000).

This paper contributes at least in two ways to the literature on civic returns to education. First, there is little evidence in the literature that separates the effect of educational attainment and cultural transmission on adult civic involvement. Second, we study the Spanish case, which has not been studied before and could have some interesting features. The teaching of citizenship has been historically absent from the Spanish non-compulsory school curricula. That is, individuals attending school after compulsory education were not generally exposed to civic education. Moreover, the Spanish compulsory school curricula did not explicitly include the teaching of citizenship until the 1990 educational reform. As suggested by Menezes (2003) or Jover and Naval (2007), after nearly forty years of dictatorship “there was an intense skepticism and fear of ideological indoctrination so any attempt to introduce civic education was intensely scrutinized and ultimately abandoned”. Hence, this might suggest that civic attitudes were mostly culturally transmitted.

This article proceeds as follows: “Data description” section presents the data and defines the variables that will be used in the analysis. In “Descriptive analysis of transmission of civic attitudes and civic awareness” section we describe the transmission of civic attitudes and civic involvement variables. “Civic returns to education and transmission of civic attitudes: empirical approach” section presents the empirical approach and in “Estimation results” section we describe the results. “Discussion and conclusions” section concludes with a discussion of the implication of these findings.

## Data description

Citizenship outcomes are examined using the Citizenship, Participation, and Democracy Survey, a Spanish national representative survey carried out by the *Centro de Investigaciones Sociológicas*, a governmental research institution (CIS 2002). The survey was collected via face-to-face interviews with a random sample of the Spanish population which included 4242 respondents aged 18 or older in the year 2002.^2^

An advantage of this survey with respect to data used in previous studies is that it includes certain questions—related to the respondent’s adolescent environment—that can be used to approximate the cultural transmission of civic values. Respondents were asked whether they recall their parents being involved in civic or political organizations, whether it was normal to discuss politics at home or at school, or if some person or event affected their way of political thinking. Similar recall questions are used by other authors who seek to analyze the transmission of family traits (Quintelier 2007; Alessina and Giuliano 2011).

Despite this advantage, as any survey regarding events or experience from the past, this survey could suffer from recall bias. This recall bias could arise from two broad sources. First, respondents might not remember some events in the past, inflating the negative answers on questions related to transmission of civic attitudes at adolescence, which might bias downwards the impact on civic involvement of transmission of civic attitudes. In order to try to mitigate this problem we select a subsample of relatively young people. Second, the questions are not asked of parents or peers of the respondent. This might imply that more (less) civically engaged respondents could more (less) likely respond that their parents were (were not) civically engaged when she was an adolescent or that their peers discussed political events at that time, etc. (e.g. cognitive dissonance). Depending on which of the two effects dominate, the civic transmission estimates might overestimate (underestimate) the true effect. Though it is not possible to empirically correct for recall bias, we consider different measures of civic transmission in order to discuss the robustness of the results.

We initially consider a subsample of individuals who turned 14 years of age between 1976 and 1990 and who were not living with their parents at the time of responding to the survey; this subsample includes 1144 observations. Spain has modified its compulsory education laws several times in the last forty years, successively increasing the compulsory school leaving age and facilitating access to post-compulsory education. This subsample includes individuals who studied under the same educational law, the 1970 General Act on Education (Ley General de Educación 1970), which established compulsory schooling up to the age of 14. Moreover, it expanded costless post-compulsory education for vocational and higher education institutions, significantly increasing the number of these institutions in Spanish regions (the number of these schools multiplied by three between 1976 and 1990).

Naturally, it could be argued that choosing a subsample of individuals subject to just one compulsory schooling regime restricts the amount of variation in education, thus potentially diminishing the influence of schooling on civic outcomes. Therefore, we increase the sample used to consider those respondents over 23 years old of age (expected to have finished their schooling) and younger than 65 (to mitigate recall bias). This new subsample increases schooling variability in two ways. First, prior to the 1970 reform, students should remain in school until age 12 (*Ley de Instrucción Pública*).^3^ Second, in 1976 child labor was reformed by increasing the minimum working age to 16 years old from 14 years of age. This modification introduced a 2-year gap between the minimum working age and the compulsory school leaving age, which was 14 years old. Hence, a would-be dropout before the labor law modification could have been induced to continue schooling, thus increasing educational attainment variability. The number of observations in this subsample is 2770.

The survey reports schooling information in two ways: as the highest educational level studied by the respondent, independently of whether she completed the degree, and as the number of full-time years of schooling. There are several facts that should be considered when using this information to characterize the impact on civic engagement of educational attainment. First, in Spain nearly 1 in every 3 individuals has at most compulsory schooling. That is, Spain is one of the OECD countries with the highest compulsory school dropout rates. Nearly 30 % of youth below 24 years of age have dropped out of the school system just after compulsory schooling leaving age (Enguita et al. 2010). Second, there is a very high repetition rate in compulsory education—nearly 50 % of 15 year old students have repeated at least 1 year during compulsory schooling—which might imply that years of full-time schooling could be a misleading measure of educational attainment.

Taking this into account, we use the survey of educational attainment information in three ways. First, we define a dummy variable for post-compulsory schooling: it takes a value of 1 if the respondent has at least some post-compulsory schooling and 0 otherwise. In this sense, we follow previous studies in this literature that analyze the impact on civic engagement of college entrance or additional schooling beyond compulsory education. Second, we define three schooling dummies, one for each school level: primary school, high school and college. Third, we define three educational attainment variables based on years of full-time schooling, one for each school level, in order to take into account the retention rate.

Below we discuss in two different subsections the variables that measure the transmission of civic attitudes—explanatory variables—and those measuring civic awareness—dependent variables.

### Transmission of civic attitudes

The survey asked the following five questions with respect to the respondent adolescence: first, *were your parents involved in civic or political organizations?* The answer takes three possible values: 1 if no parents were involved, 2 if either the mother or father were involved; 3 if the both parents were involved. Second, *was it usual to discuss politics at home?*, with four possible answers: from 1 representing not frequently to 4, frequently. Third, an identical question was asked on political discussions at school. Fourth, *was there some person who affected your way of political thinking?* And fifth, was there *some event that had affected your way of political thinking?* These last two questions take a value 1 if agreed or 0 if not.

The survey also reports the respondent’s religious engagement. It has been argued that religious involvement is correlated with civic engagement as well as with educational attainment. For example, Verba et al. (1995) find that churchgoers are more likely to be engaged in political activities. Vazquez (2007) shows descriptive evidence suggesting that in Spain there exists of a strong association between religion, educational attainment and civic awareness. Although religiosity is not our variable of interest, conditioning on this variable could be relevant because it captures intergenerational transmitted values (Guiso et al. 2003).

Furthermore, following the cultural transmission literature we have combined the above answers as vertical, horizontal and oblique transmission mechanisms dummy variables (Bisin and Verdier 2001; Bowles 2007). As *vertical transmission* we include the transmission of civic values by relatives, either through the participation in civic organizations, by discussing politics at home or by influencing the way of political thinking. There is evidence showing that family and relatives are an important source of transmission of political attitudes and behavior, reflected in adult political engagement or voting behavior (Piketty 1995; Hryscko et al. 2011).

As *horizontal transmission* we consider the political discussions at school. One possible interpretation of this variable is that it captures the explicit promotion of the teaching of citizenship issues at school. In this case, the educational attainment parameter in a civic returns equation represents a “credential effect” while the horizontal transmission parameter captures the promotion of civic attitudes at school. However, an alternative interpretation is that this variable measures pure peer interactions at school, which affects civic behavior either by sorting individuals into specific groups (teens interested and teens not interested in politics) or by conforming to the mean of the group standards (teens discussing politics because the group discusses politics). Unfortunately, form the survey information we cannot distinguish between these two alternative interpretations -school promotion or peer interaction-, relevant to assessing the effects of an explicit promotion of civic awareness in schools.

Despite this, we might expect that civically engaged parents might choose schools where teachers, peers or merely the environment makes it more inclined to discuss about politics. If this is the case, then not separating educational attainment from “discussing politics at school” might overstate the effect of formal educational on adult civic engagement.

Finally, *oblique transmission* denotes whether some particular event affected the respondent way of political thinking. Behaviors can be affected by particular political events or the environmental factors surrounding them, such as the polarizations generated by dictatorships, civil wars, poverty or democratic transitions. Conditioning on this information is relevant to understand whether the current civic involvement is enhanced by explicit actions of parents or schools or whether they are the result of pure observational learning of exogenous phenomena. If these last issues were dominant in civic involvement, then all policies aimed at promoting civic engagement could end up not being effective. We will use these last variables in the standard civic returns to education linear regression as independent variables. Additionally, we will use them to estimate a cultural transmission model in order to assess the robustness of the impact on civic engagement of the transmission of civic attitudes.

### Dependent variables: civic awareness

The civic awareness literature lacks of consensus on what entails civic engagement and how it should be measured (Campbell 2006). Here we follow McLain (2001) or Sandel (1996) and understand as a civically aware individual one who is involved in the life of their community, who is politically informed or who is actively engaged. Therefore, in order to measure civic involvement we aggregate the answers to the following questions: *discuss politics* (either at home, with friends or at work); is an *active civic participant,* participating in activities of civic associations, demonstrations, boycotts, referendums, etc.; whether the individual is *politically informed,* by newspaper readership or following political programs watched on television, followed in radio, or by online sources, and if he/she has *voted in the last election.* The answers to political discussions—at home, friends and work—runs from 1, never discuss politics, to 4, frequently discuss politics. The *active association member* counts number of times that the respondent, member of a civic or political association, has actively participated in the association activities during the last 12 months prior to the survey—for example, by volunteering, donating money, or participating in activities of the organization; similar to *demonstrations*, capturing participation (number of instances) in any type of political or civic action during the last 12 months before the survey; examples include buying fair-trade goods, participating in boycotts, signing a petition, and striking or demonstrating in person. Finally, *politically informed* measures the frequency of newspaper readership, follow-up of political programs on television, radio, or internet, ranging from 1 for never or 5 for frequently discusses politics, is engaged in organizations and/or is politically informed.

From the above questions we define two alternative measures of an active citizen: the first one is called *civic engagement intensity*, and is obtained by summing up the numerical answers given to each of the questions related to political discussions, participation in civic organization, demonstrations, political information or voting behavior, in such a way that a higher number corresponds to a more intense engagement (similar, for example, to Alessina and Giuliano 2011). The second variable, *civic engagement intensity pc (principal components)* is the first vector of the principal component analysis applied to these same questions. Both of these variables are rescaled so that its range is between 0 and 1. The correlation between these two variables is 0.97, suggesting that both provide similar information. An advantage of an aggregate measure is that treating each of the civic awareness questions separately introduces inference and interpretations problems.

In the next section we discuss some descriptive measures of the previously defined variables.

## Descriptive analysis of transmission of civic attitudes and civic awareness

As discussed in the previous section, the survey reports a series of questions that could be used to approximate the transmission of civic attitudes.

In Table 1 we present the answers given to these civic attitude transmission questions. The percentage of respondents who do not recall the answer to any of these questions is quite small: around 2 % do not recall whether their parents were involved in civic associations; slightly above 1 % do not recall politic discussions at home or at school; less than 1 % whether a particular person and nearly 4 % if a particular the event affected the way of thinking. We have recoded these answers to the worst case, assuming that not recalling implies not having being exposed to any of these issues.

**Table 1:** Transmission of civic attitude questions

Question	Percentage	Percentage		
Parents involved in civic associations				
Non of the parents	76.84			
One parent	12.50			
Both parents	10.66			
Discuss politics	At home	At school		
Frequently	9.25	6.29		
Occasionally	29.63	17.57		
Rarely	26.05	28.06		
Never	35.05	48.08		
Way of political thinking affected	Person	Event		
Yes	20.28	25.52		

Transformed civic transmission variables	Mean	Std	Min	Max
Aggregate				
Civic transmission dummy^a^	0.475	0.499	0	1
Transmission mechanisms				
Vertical transmission dummy^b^	0.368	0.482	0	1
Horizontal transmission dummy^c^	0.063	0.253	0	1
Oblique transmission dummy^d^	0.255	0.436	0	1
Active catholic	0.151	0.358	0	1

^a^Takes value 1 if parents involved in civic organizations, frequently discuss politics at home or at school, way of political thinking affected by an event or person, and zero otherwise

^b^Takes value 1 if parents involved in civic organizations, frequently discuss politics at home or way of thinking influenced by a person and 0 otherwise

^c^Takes value 1 if frequently discussed politics at school and zero otherwise

^d^Takes value 1 if the way of thinking was affected by a particular event and zero otherwise

As observed from the Table, around 1 in every 5 respondents recalls at least one of both parents having been involved in civic associations and political discussions at home or at school were scarce. Nearly 25 % state that their way of political thinking was affected by a particular person or a particular event, e.g. the democratic political transition, demonstrations, civil war etc.^4^

In the lower panel in Table 1 we present the descriptive statistics of different ways to combine these civic transmission questions in order to reduce dimensionality. We define a dummy variable, *civic transmission dummy* that takes value 1 if the respondent answered positively to at least one of the transmission questions and 0 otherwise. Nearly one in every two respondents recalled having been exposed to some type of transmission of civic attitudes. An advantage of defining this dummy variable is that it allows easy interpretation when comparing otherwise similar individuals who were and were not exposed to the transmission of civic values.

As observed form Table 1, nearly 2 of every 5 respondents recall had been exposed to vertical transmission of civic attitudes. Additionally, particularly few respondents recall frequent political discussions at school. Finally, nearly 25 % of the respondents recall that a particular event, such as the political transition after Franco’s dictatorship, affected their political way of thinking. In Table 2 we present some descriptive statistics of the variables recovering civic engagement in adulthood. Given the discrete nature of the answers, the last four columns in the table show the percentage of answers at the minimum and maximum value and the median value as well as the accumulated percentage of answers in the median.

**Table 2:** Description of the civic engagement questions

	Mean	Std	Min	Max	Distribution			
					Min^c^	Max^d^	Value^e^	Median
					%	%		%Acc^e^
Civic engagement intensity^a^	*0.23*	*0.15*	*0*	*1*	*1.66*	*0.1*	*0.2*	*50.6*
Civic engagement intensity pc^b^	*0.19*	*0.13*	*0*	*1*	*1.66*	*0.1*	*0.17*	*50.0*
Original variables								
Discuss politics^f^								
At home	2.16	0.90	1	4	27.7	6.6	2	62.9
With friends	2.21	0.93	1	4	26.7	8.1	2	60.4
At work	1.89	0.93	1	4	44.5	4.8	2	71.0
Active civic participation								
Active membership^g^	1.16	2.20	0	18	59.5	0.2	0	59.5
Demonstrations^h^	0.87	1.12	0	4	52.3	3.7	0	52.3
Political informed^i^								
Newspaper readership	2.72	1.44	1	5	27.4	17.6	2	50.0
TV political programs	2.34	1.35	1	5	34.8	12.2	2	63.4
Internet political issues	1.34	0.88	1	5	82.5	2.7	1	82.5
Voted	0.76	0.43	0	1			1	76.0

^a^Obtained by adding up all the answers given to the civic engagement questions

^b^First vector of the principal components of all civic engagement questions

^c^Percentage of answers in the minimum

^d^Percentage of answers in the maximum

^e^Value of the median; 6 % of answers accumulated at the median

^f^Response: 1 never to 4 frequently

^g^The number of different associations in which the respondent is actively participates

^h^The number of demonstrations the respondent participated in the last 12 months

Overall, as observed from Table 2, the distribution of the answers to the civic involvement questions are skewed to the left, suggesting limited civic engagement. On the one side, both of the aggregate measures have a relatively low median in terms of the range of the variable, near 0.2. On the other side, analyzing the survey questions, less than 1 in every 10 respondents discusses politics at home, with friends or at school and few actively participates in civic organizations or demonstrations. Moreover, less than a 20 % follow political issues in newspapers, by radio or online.

In the next section, and following what it is standard in this literature, we use the variables defined in the previous section to estimate a civic-returns-to-education regression including the cultural transmission variables as well as other explanatory variables which could affect current civic engagement.

## Civic returns to education and transmission of civic attitudes: empirical approach

The usual way to measure civic returns to education is by means of a linear regression of civic involvement on educational attainment, conditional on a set of explanatory variables (Dee 2004; Milligan et al. 2004; Kam and Palmer 2008; Siedler 2010; Berinsky and Lenz 2011).

In this linear model, the parameter of educational attainment measures the civic returns to education. More precisely, the parameter measures the impact on civic engagement of an increase in an individual’s educational attainment, signaling the social benefits of schooling policies, e.g., enhance the quality of democracy (as opposed to the private returns of education).

The problem, as discussed in the introduction, is that the estimator of this parameter could overstate the true civic returns to education if schooling and civic outcomes are simultaneously influenced by a variety of unobservable traits specific to the environments in which individuals are reared. In particular, it has been argued that the intergenerational or cultural transmission of civic attitudes during adolescence might be relevant in explaining both, educational attainment and adult civic behavior.

Here we estimate a linear model of civic engagement on educational attainment but, unlike previous studies, we condition on those variables capturing the cultural transmission of civic attitudes during adolescence. Our empirical strategy proceeds in two stages.

In the first stage we include in the linear model only one variable capturing transmission of civic attitudes, i.e., an aggregate measure of the transmission civic attitudes. More precisely, we aggregate all questions linked with the transmission of civic attitudes in only one variable. Similar aggregation approaches are followed in the literature, e.g., Alessina and Giuliano (2011), use aggregate measures of family ties.

Notice that no individual questions fully characterize cultural transmission of civic attitudes. Hence, when we aggregate, we increase the probability of capturing cultural transmission. Also, aggregation diminishes the sparsity problem, i.e., the significant number of zeroes in the intersection of questions and characteristics, that might affect the precision of the parameter estimators; or the problem of multicollinearity that appears as we include different variables capturing transmission, as individuals that positively respond one question related to civic engagement tends to positively respond similar questions.

In sum, in the first stage we study whether any type of active transmission of civic attitudes during adolescence enhances civic engagement during adulthood.

In the second stage, we discuss the relative importance of the different transmission mechanisms. That is, we decompose cultural transmission into the three mechanisms discussed in the cultural transmission literature (e.g., Bisin and Verdier 2010). Vertical transmission is associated with the transmission of civic attitudes by relatives; this transmission may be either through participation in civic organizations, by discussing politics at home or influencing the way of political thinking. Horizontal transmission, which in this case capture political discussion at school during adolescence. As stated before, this variable could be recovering the explicit promotion of the teaching of citizenship at school or peer interactions at school. Finally, oblique transmission considers whether some particular event affected the respondent way of political thinking.

## Estimation results

In Table 3 we present the OLS estimates of civic returns to education equation where we consider a unique variable capturing the transmission of civic attitudes. In columns I and V we present the estimated coefficients not including the transmission of civic attitudes variables. In the subsequent columns we include controls for the transmission of civic attitudes: in columns II and VI, we include a single variable, the civic transmission dummy; in columns III and VII we additionally include the interaction term between educational attainment and civic transmission. Finally, in columns IV and VIII we increase the sample size to capture greater educational attainment variability. To isolate the effect of educational attainment and transmission of civic attitudes from other possible confounding effects we control for variables representing basic demographic information on age, gender, marital status; variables to control for the opportunity cost of time are: family income, full-time work, partner working full-time, civil servant; we additionally introduce state and province fixed effects, urbanicity (dummies for size of residence city); finally, we include the percent of parents -old generation- with a high school diploma, in order to capture environmental factors that might strengthen schooling and civic commitment.

**Table 3:** OLS civic returns to education including transmission of civic values

	Dependent variable							
	Civic engagement intensity (add-up answers)				Civic engagement intensity (principal components)			
	I	II	III	IV	V	VI	VII	VIII
Post-compulsory schooling (PCS)	0.075***	0.062***	0.047***	0.041***	0.062***	0.054***	0.037***	0.034***
	(0.016)	0.015	(0.015)	(0.01)	(0.014)	(0.013)	(0.012)	(0.008)
Active catholic	0.048***	0.047***	0.047***	0.023***	0.044***	0.044***	0.044***	0.022***
	(0.012)	(0.012)	(0.012)	(0.007)	(0.01)	(0.01)	(0.011)	(0.006)
Civic transmission dummy (CT)		0.093***	0.067***	0.058***		0.083***	0.061***	0.053***
		(0.01)	(0.014)	(0.005)		(0.007)	(0.012)	(0.004)
PCS*CT			0.038**	0.034***			0.033**	0.034***
			(0.014)	(0.006)			(0.013)	(0.006)
No observations	1144	1144	1144	2770	1144	1144	1144	2770

Regressions include cohort dummies, gender, married, region dummies, income, working full time, partner working full-time and size of the city dummies. Results based on weighed regressions and robust clustered by county standard errors. The *p* value of overall significance of the model is zero in all cases

***1 % significance level; **5 % significance level; *10 % significance level

Overall, the above estimates suggest that the influence on civic engagement of additional schooling and of the transmission of civic awareness is both positive and statistically significant. This result is consistent with previous findings in the literature and with the expected belief that intergenerational transmission of civic attitudes should be positively correlated with civic engagement in adulthood. In addition, it implies that civic returns to education could be overestimated if the transmission of civic attitudes were omitted from the civic returns to education regression.

Nevertheless, to our understanding the most appealing result implied by the above estimates is the impact of cultural transmission of civic attitudes on civic engagement in adulthood. First, note in columns II and VI that the transmission of civic attitudes point estimates of are nearly fifty percent larger than the educational attainment point estimates: civic involvement is increased by 0.09 (0.08) percentage points for individuals exposed to transmission of civic attitudes and by 0.063 (0.051) percentage points as a consequence of additional schooling. That is, civic engagement in adulthood seems to be more responsive to the transmission of civic attitudes in adolescence than to additional schooling.

Second, columns III (IV) and VI (VII) show that the transmission of civic attitudes significantly reinforces the role of education in enhancing civic engagement: the interaction term between educational attainment and transmission of civic values is positive and statistically significant. The estimate of civic returns to education is around 0.15 for individuals exposed to cultural transmission of civic attitudes and 0.04 for those not exposed. In other words, individuals exposed to intergenerational transmission of civic values are four times more likely to be civically engaged in adulthood than those not exposed, all else equal.

In Table 4 we repeat the above exercise but consider different educational attainment variables. In columns I and II educational attainment is characterized by three dummy variables that capture the different educational levels in the Spanish schooling system: elementary school (compulsory school), high school (which includes vocational school) and college; in columns III and IV we use years of full time education conditional on the highest educational level studied.

**Table 4:** Civic returns to education with different definition of educational attainment

Dependent variable civic engagement intensity (principal components)					
	I	II		III	IV
Elementary school (PS)	0.048**	0.048**	Years school ES	0.0026***	0.0025***
	(0.020)	(0.020)		(0.0010)	(0.0010)
High school education (HSE)	0.081***	0.074***	Years school HSE	0.0040***	0.0034***
	(0.022)	(0.024)		(0.0004)	(0.0004)
College Education (CE)	0.135***	0.111***	Years school CE	0.0057***	0.0044***
	(0.028)	(0.029)		(0.0004)	(0.0004)
Civic transmission dummy (CT)	0.068***	0.054***	CT	0.069***	0.056***
	(0.003)	(0.004)		(0.003)	(0.004)
HSE*CT		0.018***	YSHSE*CT		0.0011***
		(0.006)			(0.0004)
CE*CT		0.042***	YSCE*CT		0.0022***
		(0.010)			(0.0006)
No observations	2770	2770		2770	2770

The p-value of overall significance of the model is zero in all cases

***1 % significance level; **5 % significance level; *10 % significance level

A first interesting result observed in the above estimates is the nonlinear impact of educational attainment on civic involvement. For those with a college degree but not exposed to the transmission of civic attitudes the impact is around 0.135 (0.111) compared to 0.048 (0.048) of those with only elementary schooling (e.g. compulsory schooling; cols. I and II). Or, the impact of an additional school year for those who only achieved elementary school is 0.0026 (0.0025) compared with 0.0057 (0.0044) for those with a college degree but not exposed to civic transmission. The difference between these estimates are statistically significant, i.e. there is a statistically significant increase in the impact of additional schooling when moving from primary school to high school or to college: individuals not exposed to the transmission of civic attitudes are three times more likely to be civically engaged if attended college than those who only attended primary school.

Second, the qualitative results of Table 4 on civic returns to education regressions are similar to those presented before. Despite the inclusion of the transmission of civic attitudes, educational attainment is positively and strongly associated with civic engagement. In addition, the transmission of civic attitudes significantly reinforces the impact on civic engagement of education attainment. The interaction effect between college educational attainment and transmission of civic attitudes increases civic returns to education by nearly a 50 % of its reference impact and by nearly a third for high school educational attainment.

Overall, the results above might be suggestive of two issues based on the positive and statistically significant interaction effect between educational attainment and the transmission of civic attitudes. The first one refers to a possible sorting towards higher educational levels depending on the transmission of civic attitudes. That is, taking into account that it is more likely that those individuals reared in environments that stress civic attitudes are encouraged to attain higher educational levels, then the transmission of civic attitudes could be a sorting mechanism towards post-compulsory schooling. In this sense, this could imply that the better-educated individuals will tend to reproduce or promote their particular viewpoints if the educational system does not expose students to alternative points of view, as argued by civic virtue theories.

An second empirical issue suggested by the results above is concerned with the separability assumption when the transmission of civic attitudes is not observed. That is, the statistical significance of the interaction term between schooling and the transmission of civic attitudes can imply that the partial response of the civic outcomes from changing the schooling level depends on the level of the unobservable confounders (in those studies where the transmission of civic attitudes is not observed).

In Table 5 we present the OLS estimates from when we decompose the transmission of civic attitudes into its different mechanisms. We use different educational attainment variables: in column I a dummy variable captures post-compulsory schooling; in column II, dummies for each educational level and in column III the number of full-time school years depends on the educational level attained.

**Table 5:** Civic returns to education with different definition of educational attainment and transmission of civic attitudes

Dependent variable civic engagement intensity (principal components)					
	I		II		III
Post-compulsory schooling (PCS)	0.032***	Elementary school (ES)	0.050***	Years school ES	0.0025***
	(0.007)		(0.017)		(0.0010)
		High school education (HS)	0.076***	Years school HS	0.0034***
			(0.021)		(0.0003)
		College education (CE)	0.111***	Years school CE	0.0042***
			(0.027)		(0.0004)
Vertical transmission dummy (VT)	0.036***	VT	0.036***	VT	0.034***
	(0.005)		(0.004)		(0.004)
Horizontal transmission dummy (HT)	0.021*	HT	0.030***	HT	0.028***
	(0.011)		(0.007)		(0.007)
Oblique transmission dummy (OT)	0.054***	OT	0.058***	OT	0.059***
	(0.006)		(0.004)		(0.004)
PCS*VT	0.010**	HS*CT	0.008	YSHS^CT^1^	0.0006
	(0.005)		(0.008)		(0.004)
PCS*HT	0.017*	CE*CT	0.025**	YSCE*CT^2^	0.0015**
	(0.010)		(0.011)		(0.0006)
PCS*OT	0.019***				
	(0.007)				
No observations	2770		2770		2770

Regressions include cohort dummies, gender, married, region dummies, income, working full time, partner working full-time, size of the city dummies. Results based on weighed regressions and robust clustered by county standard errors; 1 *YSHS* (years schooling high school maximum educational attainment) interacted with civic transmission; 2 *YSCE* (years schooling college maximum educational attainment)

The p-value of overall significance of the model is zero in all cases

***1 % significance level; **5 % significance level; *10 % significance level

Overall, these estimates suggest that the impact on civic involvement of the three transmission mechanisms as well as educational attainment is positive and significant in all regressions. Moreover, the interaction effects between education level and transmission of civic attitudes are positive and significant, implying that the transmission of values reinforces the impact of education on civic involvement.

A first interesting result in the above regressions is that the horizontal transmission mechanism has a positive and significant impact on civic involvement. That is, political discussions at school when an adolescent is positively associated with an increase in adult civic involvement. Notice that if this variable is approximating some sort of teaching of citizenship where students discussed politics, then this estimate might be suggesting that the contents (i.e. what people actually learn) could help to shape civic involvement.

A second appealing result in the above estimates is the suggestion that oblique transmission seems to be the most relevant mechanism in enhancing adult civic engagement. That is, having been exposed to particular events significantly increases civic involvement, much more than vertical or horizontal transmission. Hence, those cohorts of individuals which experienced events that shaped their political way of thinking will be more likely to be civically engaged in adulthood than those not exposed, all else equal.

Finally, the impact on civic involvement of the vertical transmission of civic attitudes is also positive and significant. That is, the transmission of values by parents and relatives during adolescence has a positive impact on civic involvement in adulthood.

## Discussion and conclusions

For some political, social and educational theorist, the stability of democracy depends in great extent on the qualities and attitudes of their citizens (e.g., Kymlicka 2002; Kymlicka and Norman 1994; Habermas 2010, 1996). In sum, there is a belief that without an active citizenship, liberal societies are difficult to govern in the common good (Sandel 2010).

However, since the postwar period until around the nineties of last century the passive concept of citizenship was mostly the predominant paradigm. Citizenship was conceived as the passive entitlement of rights, guaranteed by the welfare state and with the absence of any obligation to participate in public life. In European welfare states, individuals were conceived as rights-claimers, where the government assumed a paternalistic role which drove citizens to increase passiveness (Kymlicka and Norman 1994; Roche 1992).

Now, in the turn of the new century, not few democracy advocates have been arguing that the passive model of citizenship which inspired educational and political institutions have lead to certain undesired civic outcomes that could endanger the sustainability of democracy. For example, an increasing voter apathy and a general political disenchantment; the perception of a loss of civic attitudes or behaviors, such as greater intolerance in multicultural societies; the diminishing engagement in social or civic organization, observed in a dramatic fall in unionism, etc.

This evidence raised a renewed debate on citizenship virtues in democratic societies and the role of schools (Beck 2006; Putman 2001; Lockyer et al. 2003; Stolle and Hooghe 2004; Kiss and Euben 2010).

For liberal theorists, who prime individual’s autonomy, civic and political commitments are optional and are as valid as any other way of life freely chosen, i.e. “citizenship is a minimal legal status largely fulfilled by paying taxes and obeying the law” (Kiss and Euben 2010). For them, the above evidence shows no more than the outcomes of deliberative processes freely engaged by autonomous individuals in democratic societies. Even more, some argue that the neutral position adopted by the schooling system in terms of the teaching of citizenship or civic morality has been effective in educating civic values given the significant positive correlation between higher levels of schooling and different measures of civic behavior, i.e., civic socialization is the result of schools own social dynamic (Caplan 2006).

On the other extreme, civic republicanism or communitarianism theorists consider that individuals should serve the common good—engagement in public or community affairs is privileged over personal interests—and view the above facts as a loss of civic attitudes or values, which at the end, could endanger democracy sustainability. For these theorists, is not in school where individuals learn civic attitudes but actively engaging in public affairs or in any associational network in civil society.

In the middle of these two extremes, civic virtue theorists consider that the above facts show some type of flaw in the way citizenship values are being transmitted to younger generations. They disagree with republicanism or communitarianism theorists on the way civic values are acquire, in the sense that people do not automatically learn how to engage in political or civil society associations, question authority or construct a critical thinking about public issues. For them, schools are the ones who have to teach children how to engage in critical thinking about the status quo, about political affairs, about the reasonableness of civic attitudes or behaviors to sustain democracies, etc. (Nussbaum 2010; Gutmann 1999; Kymlicka and Norman 1994).

And civic virtue theorist disagrees with liberal theorists on whether the academic educational system is effectively transmitting civic virtues. For civic virtue theorists, the strong correlation between schooling and civic attitudes -and which to some extent supports the argument of liberal theorists for a neutral civic value curricula- could occur because the children of politically aware or civically engaged parents could be more likely to stay in school and adopt their parents civic values or concept of good. And, if this were to be the case, then the passive citizenship schooling system is not adequately transmitting civic virtues.

Taking into account the previous discussion, in this empirical paper we seek to shed some light on the separate influence of the transmission of civic attitudes and of educational attainment on civic engagement using Spanish data. Unlike most of the previous studies on this issue, we are able to approximate the transmission of civic attitudes during adolescence.

Overall, our findings suggest that the transmission of civic attitudes during adolescence has a strong and positive association with civic engagement in adulthood. Moreover, the schooling effect is amplified by the transmission of civic attitudes, in the sense that the interaction terms are positive and significant. In other terms, it seems as if those parents or relatives that transmit civic attitudes also encourage their children to more schooling. Hence, if schools do not transmit civic values, these children will carry the view transmitted by their parents or relatives.

From a policy point of view, our findings support the view of those advocates who suggest that civic values should be taught at schools. As our results suggest, cultural transmission of civic values plays an important role in civic engagement. Hence, it seems relevant that a democratic state promotes other views or values within the formal schooling system.

## Footnotes

1 Even in higher education, teaching professional ethics has been replaced by more technical teaching (Callan 1997; Flanagan et al. 2008; Kiss and Euben 2010).

2 The CIS has run other surveys analyzing political engagement (2005, 2006 and 2009) but there is no continuity on the questions nor the target population.

3 The individuals considered in our subsample were not affected by the 1990 LOGSE educational reform.

4 In two of every three cases, the person that influenced the respondent way of political thinking was a relative (CIS 2002, Survey 2818).
